# Draft genomes of *Aeromonas caviae* from patients with cholera-like illness during the 2022–2023 cholera outbreak in Malawi

**DOI:** 10.1128/MRA.00580-23

**Published:** 2023-09-28

**Authors:** Khuzwayo C. Jere, Innocent Chibwe, David Chaima, Watipaso Kasambala, Chimwemwe Mhango, Joseph Bitilinyu-Bangoh, Bernard Mvula, Wakisa Kipandula, Arox W. Kamng'ona, A. Duncan Steele, Annie Chauma-Mwale, Daniel Hungerford, Matthew Kagoli, Martin M. Nyaga, Queen Dube, Neil French, Nigel A. Cunliffe, Chisomo L. Msefula, Chrispin Chaguza

**Affiliations:** 1 Department of Clinical Infection, Microbiology and Immunology, Institute of Infection, Veterinary and Ecological Sciences, University of Liverpool, Liverpool, United Kingdom; 2 Malawi-Liverpool-Wellcome Research Programme, Kamuzu University of Health Sciences, Blantyre, Malawi; 3 Department of Medical Laboratory Sciences, Faculty of Biomedical Sciences and Health Profession, Kamuzu University of Health Sciences, Blantyre, Malawi; 4 NIHR Global Health Research Group on Gastrointestinal Infections, University of Liverpool, Liverpool, Merseyside, United Kingdom; 5 National Microbiology Reference Laboratory, Public Health Institute of Malawi, Lilongwe, Malawi; 6 Department of Pathology, School of Medicine and Oral Health, Kamuzu University of Health Sciences, Blantyre, Malawi; 7 Department of Biomedical Sciences, School of Life Sciences and Allied Health Professions, Kamuzu University of Health Sciences, Blantyre, Malawi; 8 Diarrhoeal Pathogens Research Unit, Sefako Makgatho Health Sciences University, Medunsa, Pretoria, South Africa; 9 Next Generation Sequencing Unit and Division of Virology, Faculty of Health Sciences, University of the Free State, Bloemfontein, Free State, South Africa; 10 Malawi Ministry of Health, Lilongwe, Malawi; 11 Department of Epidemiology of Microbial Diseases, Yale School of Public Health, YaleUniversity, New Haven, Connecticut, USA; 12 Yale Institute for Global Health, Yale University, New Haven, Connecticut, USA; 13 NIHR Mucosal Pathogens Research Unit, Research Department of Infection, Division of Infection and Immunity, University College London, London, United Kingdom; 14 Parasites and Microbes Programme, Wellcome Sanger Institute, Hinxton, United Kingdom; Loyola University Chicago, Chicago, Illinois, USA

**Keywords:** *Aeromonas caviae*, sub-Saharan Africa, cholera-like illness, whole-genome sequence, cholera outbreak, Malawi

## Abstract

*Aeromonas caviae* is an increasingly recognized etiological agent of acute gastroenteritis. Here, we report five draft genomes of *A. caviae* isolated from suspected cholera cases during the 2022–2023 cholera outbreak in Malawi.

## ANNOUNCEMENT

*Aeromonas* species cause a range of clinical infections, including gastroenteritis, bacteremia, septicemia, peritonitis, pneumonia, and wound infections (1). Several *Aeromonas* species have been identified, of which *A. caviae*, *A. hydrophila*, *and A. veronii* are the most clinically significant. Several studies in Africa and elsewhere have reported the isolation of *A. caviae* in patients presenting with cholera-like symptoms of profuse watery diarrhea and vomiting (2–6). However, no systematic population genomic studies of *A. caviae* have been reported from cholera-endemic regions. In this report, we announce draft whole-genome sequences of five *A. caviae* isolates collected from patients with suspected cholera during the 2022–2023 cholera outbreak in Malawi (7), the largest and deadliest cholera outbreak in the country’s history (8, 9).

The *A. caviae* sequences described in this report were obtained following the whole-genome sequencing of bacterial isolates that, following growth on selective media, were provisionally suspected to be *Vibrio cholerae*. Detailed descriptions of the laboratory methods used to culture the isolates were described previously (7). Genomic DNA was extracted using QIAamp DNA Mini kit (Qiagen, Germany) and quantified on a Qubit fluorometer using a High Sensitivity dsDNA Assay kit (Thermo Fisher Scientific, USA). Genomic libraries were prepared at the UFS-NGS Unit using the Nextera XT DNA Library preparations kit (Illumina, USA). The quality of the libraries and fragment size distribution was assessed on Agilent 2100 Bioanalyzer using the dsDNA High Sensitivity Assay kit (Agilent Technologies, USA), and the average fragment size obtained was 600 bp. DNA sequencing was performed on a MiSeq platform (Illumina) for 600 cycles, using a V3 reagent kit (Illumina, USA) to generate 2 × 301 pb paired-end reads (7). Of 68 isolates suspected to be *V. cholerae* following provisional biochemical identification, five (uncontaminated) were identified as *A. caviae* by sequencing.

**Fig 1:**
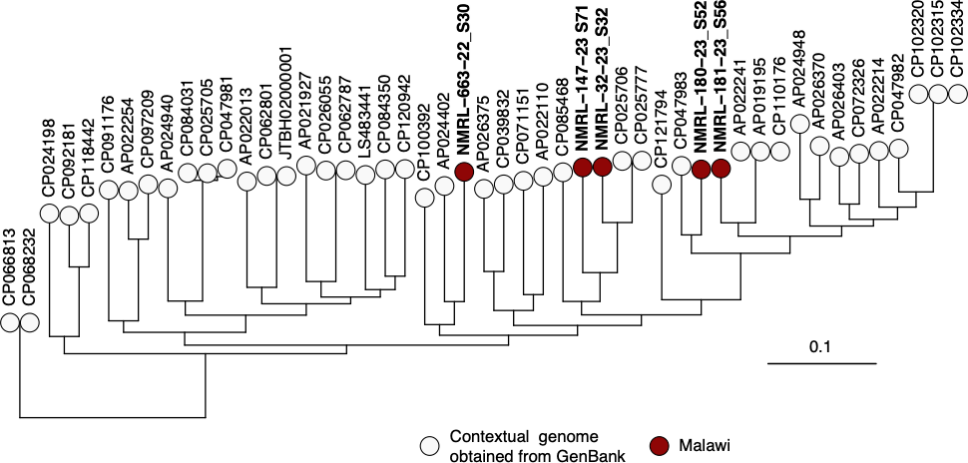
Maximum likelihood phylogeny of *A. caviae* isolated from cholera-like illness in Malawi in the context of the publicly available genomes in the GenBank database. The phylogeny was generated based on 383,314 SNPs using FastTree. The tree was rooted on the branch separating the *A. caviae* genomes from the outgroup sequence for *A. sanarellii* strain MaGu-431 (GenBank assembly accession: GCF_017815105). The taxon labels for the *A. caviae* genomes from Malawi are shown in bold text in the phylogeny. For clarity, the outgroup is not shown in the phylogenetic tree.

The raw sequencing reads were trimmed using cutadapt v4.4 (10). The species assignment and removal of human reads were done using Kraken v2.1.2 (11). Only a minority of the reads (<1%) were classified as non-*Aeromonas* species. The non-human reads were assembled using SPAdes v3.14.0 (12). Sequence types (STs) of the *A. caviae* were identified by scanning the assemblies against PubMLST typing schemes (13) using mlst v2.19.0 (https://github.com/tseemann/mlst). The reads of *A. caviae* isolates from Malawi and those obtained from GenBank were mapped to the *A. caviae* reference genome strain 8LM (GenBank accession: CP024198) (14) using snippy v4.6.0 (https://github.com/tseemann/snippy) to generate pseudo-whole-genome alignments. Single nucleotide polymorphisms (SNPs) were identified in the alignment using snp-sites v2.5.1 (15) and used to generate a maximum likelihood phylogeny using FastTree v2.1.10 (16) (Fig. 1). The average read length was assessed using seqkit v2.4.0 (17). Coding sequences were determined using NCBI Prokaryotic Genome Annotation Pipeline v6.5 (18). Genome completeness and contamination statistics were assessed using checkM v1.2.2 (19). The reads and assembly characteristics are summarized in Table 1.

**TABLE 1:** Summary of the assembly details and statistics

Isolate	NMRL-147-23_S71	NMRL-180-23_S52	NMRL-181-23_S56	NMRL-32-23_S32	NMRL-663-22_S30
Species	*Aeromonas caviae*	*Aeromonas caviae*	*Aeromonas caviae*	*Aeromonas caviae*	*Aeromonas caviae*
Average nucleotide identity (%)	97.94	97.97	98.06	97.92	97.81
ST	Novel	Novel	Novel	Novel	Novel
Source	Human stool	Human stool	Human stool	Human stool	Human stool
Isolation date	09 February 2023	09 February 2023	09 February 2023	10 January 2023	24 October 2022
NCBI BioSample accession	SAMN35977324	SAMN35977325	SAMN35977326	SAMN35977327	SAMN35977328
NCBI BioProject accession	PRJNA987340	PRJNA987340	PRJNA987340	PRJNA987340	PRJNA987340
Genome assembly accession^*a*^	JAUDRR000000000	JAUDRS000000000	JAUDRT000000000	JAUDRU000000000	JAUDRV000000000
GenBank assembly	GCA_030412495.1	GCA_030412505.1	GCA_030412535.1	GCA_030412565.1	GCA_030412555.1
NCBI Sequence Read Archive accession	SRR25017219	SRR25017218	SRR25017217	SRR25017216	SRR25017215
Assembly length (bp)	4,381,030	4,245,009	4,568,182	4,560,284	4,256,869
No. of contigs	568	474	892	869	532
N50 (bp)	22,466	21,959	13,362	17,279	20,319
L50 (bp)	62	55	101	75	53
Mean contig length (bp)	7,713.08	8,955.72	5,121.28	5,247.738	8,001.63
Longest contig (bp)	78,530	120,788	51,172	75,134	146,958
G+C content (%)	61	61	60.5	61	61
No. of sequence reads	198,449	205,100	210,377	225,665	236,299
Read type	Paired	Paired	Paired	Paired	Paired
Average read lengths (bp)	242.15	235.4	234.4	223.5	225.15
Sequencing method	Illumina MiSeq	Illumina MiSeq	Illumina MiSeq	Illumina MiSeq	Illumina MiSeq
Genome coverage	80.5×	82.8×	80.7×	81.9×	81.4×
Completeness (%)	97.03	92.9	95.97	95.08	94.18
Contamination (%)	0.99	0.34	1.6	2.14	1.1
No. of genes	4,395	4,235	4,805	4,780	4,326
No. of protein-coding genes	3,994	3,851	4,189	4,220	3,914
No. of non-coding genes	136	155	150	148	144

^*a*^ Draft genome assemblies submitted were trimmed to remove adapter sequences and other potential contaminations. Only contigs >200 bp were included in the sequences submitted to NCBI GenBank.

## Data Availability

This whole-genome shotgun project has been deposited at GenBank under the accession numbers in Table 1.
